# Quantitative Analysis of Defects at the Dentin-Post Space in Endodontically Treated Teeth

**DOI:** 10.3390/ma8063268

**Published:** 2015-06-04

**Authors:** Mariasevera Di Comite, Vito Crincoli, Laura Fatone, Andrea Ballini, Giorgio Mori, Biagio Rapone, Antonio Boccaccio, Carmine Pappalettere, Felice Roberto Grassi, Angela Favia

**Affiliations:** 1 Department of Basic Medical Sciences, Neurosciences and Sensory Organs, University of Bari, Piazza G. Cesare, 11, Bari 70124, Italy; E-Mails: andrea.ballini@uniba.it (A.B.); angela.favia@uniba.it (A.F.); 2 Interdisciplinary Department of Medicine, University of Bari, Piazza G. Cesare, 11, Bari 70124, Italy; E-Mails: vito.crincoli@uniba.it (V.C.); laura.fatone@gmail.com (L.F.); biagiorapone@virgilio.it (B.R.); feliceroberto.grassi@uniba.it (F.R.G.); 3 Department of Biomedical and Experimental Sciences, University of Foggia, Viale Luigi Pinto, 1, Foggia 71122, Italy; E-Mail: giorgio.mori@unifg.it; 4 Department of Mechanics, Mathematics and Management, Polytechnic of Bari. Viale Japigia, 182, Bari 70126, Italy; E-Mails: a.boccaccio@poliba.it (A.B.); carmine.pappalettere@poliba.it (C.P.)

**Keywords:** endodontic cements, irrigants, endoposts, adhesive defects, morphometric analysis

## Abstract

The objective of this study was to assess frequency and extension of the defects affecting the dentin-post interface after using different combinations of irrigants and sealers. The experimental work was conducted on single-rooted teeth extracted for orthodontic reasons. The specimens were divided into different groups, according to irrigant and endodontic cement utilized, and endodontically instrumented. After fiberglass posts cementation, cross sections were obtained at apical, middle and coronal level of the root and submitted to quantitative analyses. Different types of defects were found: bubbles, bonding defects, polymerization defect, and cement residues. The percent extension of each defect and its frequency were related to the specific irrigant/sealer combination and to the root level. Detachments of the material from dentin were found only at apical and middle levels. Chlorhexidine digluconate seems to have more beneficial effects if compared to sodium hypochlorite: samples prepared with chlorhexidine digluconate showed a higher performance, with roots including null to few defects. In detail, samples treated with chlorhexidine digluconate and Pulp Canal Sealer showed the lowest frequency and the smallest dimension of defects.

## 1. Introduction

Endodontically treated teeth run a higher risk of mechanical failure in comparison to vital ones. The major cause of fracture can be attributed to the loss of dental substance because of caries or cavity preparation [1,2,3]. Furthermore, the loss of the physiologic vascularization and hence the water loss or the reduction of collagen cross-linking could make the endodontically treated teeth more brittle [4,5].

Endodontic posts are necessary for the retention of the coronal restoration, though they do not increase the mechanical resistance of the dental roots. To reduce the risk of non-recoverable root fracture, posts with mechanical properties more similar to the dentinal tissue have been introduced [3,6], thus replacing the traditional metallic posts [7,8]. Since fiber posts have a Young’s modulus close to dentinal tissue, the stress state at the dentin-post interface tends to distribute homogeneously. For this reason, fiber posts reduce the risk of root fractures rather than metallic ones [9,10,11,12,13]. Other advantages of fiber posts are the adhesive bonding technique, which requires minimal intervention on the dentinal surface [14], a restoration procedure that can be conducted in a shorter time with relatively low costs [15,16], and the aesthetic outcome [13].

The debonding of the fiber post from its post-space has been shown to take place principally between adhesive luting material and dentin [17]. This may be due to different factors, such as insufficient polymerization of adhesive materials along the canal, high polymerization shrinkage [18], unfavorable C factor [19], negative influences of intracanalar irrigating solutions and endodontic sealers [20], type of dentinal tissue [21], and presence of residual pulpal tissue [22].

Chemical irrigants are essential for successful debridement of root canals during shaping and cleaning procedures [22,23,24,25,26,27]. Sodium hypochlorite (NaOCl) dissolves organic tissues and neutralizes toxic products, whereas chlorhexidine digluconate (CHX), in addition, has bactericide properties [28]. Irrigants may also affect the characteristics of the dentinal tissue. For instance, NaOCl makes oxidized dentin, which inhibits the process of adhesion of resinous materials to dentin or their polymerization [2,29,30,31,32,33]. CHX inhibits matrix metalloproteinases, thus increasing the adhesion of luting agents with dentin [34,35].

Regarding endodontic sealers, the persistency of residues on the root canal walls could cause defects within the post-core materials, thus affecting the bond strength [36,37,38,39,40,41].

In spite of a huge number of studies on the effects of chemical irrigants and endodontic sealers on the interfacial strength, poor information is available regarding to the quantitative defects caused by different irrigant/sealer combinations. In a previous work [42], the effects of different irrigating solutions and endodontic sealers on mechanical strength of the dental restoration were investigated. The novelty of the present study is based on the relationship between the most representative defects at the dentin-post space and the irrigant/sealer combination.

The aim of this research was to analyze the defect distribution, their frequency and their percent extension on the different levels of the dental root. The null hypothesis was that the combination irrigant/sealer does not affect the values of the interfacial defects.

## 2. Experimental Section

### 2.1. Preparation of Samples

Forty-eight human single-rooted teeth were extracted for orthodontic reasons and collected for this study. Eight of them were then excluded because they had fractured during surgical procedures. None of the remaining forty teeth had received previous restorative or endodontic treatment. Following the same protocol adopted in a previous investigation [42], teeth were kept in 1% chloramine T (pH 7.8) at 4 °C until use [43]. With a high-speed carbide bur and water spray, teeth were sectioned below the cement-enamel junction, perpendicularly to the long axis, obtaining approximately 15 mm long root segments.

The roots were randomly assigned to four groups (10/group) according to the irrigating solution (NaOCl or CHX) as well as to the type of endodontic cement, Pulp Canal Sealer™ (EWT KERR^®^, Orange, CA, USA) or Apexit Plus (IVOCLAR VIVADENT^®^, Naturno, Italy).

Canal patency and working length were established by inserting #10/02, #15/02 and #20/02 tapered K file (DENTSPLY Maillefer, Ballaigues, Switzerland) to the root canal terminus. The canals were prepared at working length using Pro Taper instrument S1, S2 and F1 (DENTSPLY Maillefer, Ballaigues, Switzerland) according to Ruddle’s protocol [44]. After using 20/07 F1 Pro Taper instrument, the size of the foramen was gauged with a# 20/02 tapered hand file: the #20 hand file was snug and the canal was considered fully shaped [44]. In order to remove the smear layer left by every file, they were abundantly irrigated with 0.2% CHX (groups A and C) or 5.25% NaOCl (groups B and D).

After chemical-mechanical preparation, canals were dried using paper points.

Pulp Canal Sealer (PCS) cement based on zinc oxide and eugenol, was used for group A and B, while Apexit Plus (AP) cement based on calcium hydroxide, was used for group C and D (Table 1).

**Table 1:** Endodontic cement/irrigant combinations utilized in the present study.

Irrigants	Pulp Canal Sealer™ EWT KERR^®^	Apexit Plus IVOCLAR VIVADENT^®^
CHX	group A	group C
NaOCl	group B	group D

System B pluggers, size fine/medium were utilized to condense the gutta-percha master cone to within 5 mm from the working length. Delayed cementation of fiber post (at least 24 h after post endodontic treatment) resulted in higher retentive strengths in comparison to immediate cementation [45]. After storage at 100% humidity for 1 week at 37 °C, a post-space was created and the excesses of gutta-percha and sealers were removed by means of Gates-Glidden burs n° 3 (DENTSPLY Maillefer-Ballaigues, Switzerland), maintaining at least 4–5 mm of apical seal. Then, root canals were once again abundantly irrigated with 0.2% CHX (groups A and C) or 5.25% NaOCl (groups B and D). The same irrigants used in the chemical-mechanical preparation of the specimen were then used in the post-space irrigation. Paper points were again utilized to dry their surface.

Fiberglass posts were inserted according to the protocol prescribed by the posts’ Manufacturer (MC Italia, Milano, Italy). Using a Surgi Shaper cutter disk, the post was adapted apically. Before cementation, each fiber post was cleaned with 70% ethanol and dried.

According the manufacturer’s instruction, the root canal walls were treated with phosphoric acid 37% for sixty seconds. The acid was then removed by distilled water. Paper points were again used, leaving the still wet dentinal surface. By using an extra-fine microbrush, a first layer of dual adhesive Surgi Primebond Base + Surgi Primebond Activator was applied on the canal walls. Then, the layer was radiated by an LED lamp (Mini LED SuperCharged, Financiere Acteon SAS, Merignac Cedex, France) for twenty seconds. According to the Manufacturer’s instructions, a second layer of dual adhesive was applied but not radiated to avoid the formation of an additional thickness that would shift the post in the coronal direction. The dual adhesive was also applied on the post surface. The dual cement filled the root canal completely by means of auto-mix endodontic tips. The post was finally inserted and the excessive amounts of cement that came out of the root canal were radiated for forty seconds to stabilize the emergent part of the post. The light output of the LED-curing unit was monitored by a radiometer. All the operations were carried out by a single experienced practitioner.

### 2.2. Quantitative Analysis

The endodontically treated roots were fixed in 70% ethanol for two days at 4 °C, dehydrated and conventionally processed for metyl-methacrylate embedding. Serial sections (80 μm thick) were obtained according to a transverse plane by means of a circular diamond-bladed saw microtome (SP1600 Leica Microsystems, Wetzlar, Germany) and thinned up to 70 μm under running water on fine-grain sandpaper. Their final thickness was checked by means of a micrometric caliper and then air dried in incubator at 37 °C for 24 h.

**Figure 1:**
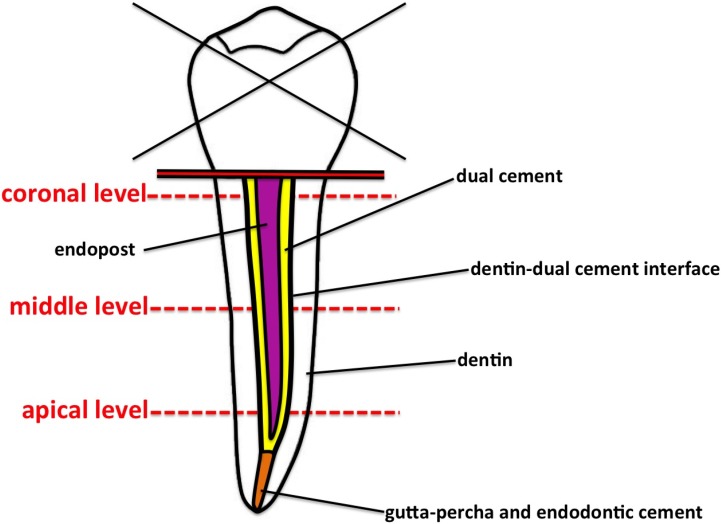
Schematic drawing of the endodontically treated root showing the three levels analyzed.

In order to search possible anomalies present along the inner walls of endodontic space, one section at apical, middle and coronal level of the post was chosen (Figure 1). The specimens were observed under a light microscope (Nikon Eclipse E 400, Nikon Corporation, Tokyo, Japan) equipped with a digital camera (Nikon DS-5M) at 100× enlargement. Five different types of defects were found:

(a) inclusion of gas bubbles within the dual cement layer (bubbles);
(b) bonding defects at the dentin-dual cement interface (detachments);
(c) nebulous-looking areas within the dual cement layer due to a polymerization defect (pol.def.);
(d) endodontic cement residues at dentin–dual cement interface (int.res.);
(e) embedded residues of endodontic cement within the dual cement layer (emb.res.).

About point c, three sections selected into each group were submitted to microhardness test, according to Vickers (HV), to state if the nebulous-looking areas are due to curing abnormalities. These anomalous polymerized regions show lower HV values than normal ones [46]. The sections were processed in accordance with a previously standardized procedure [47]. HV was tested with a Leitz Durimet by applying a square-base pyramidal diamond indenter (Vickers pyramid with angles of 136°) under a load of 25 g. The HV was computed from the formula HV = 1854.4 × L/D2, where L is the load in [g] and D the mean length in μm of the imprint’s diagonals measured at an enlargement of 400×. HV values resulted always lower in the nebulous than in the normal-looking adjacent regions.

Furthermore, the following evaluations have been carried out:

(a) the frequency* of each type of defect computed both taking all the sections together, both taking into account the specific levels;
(b) the frequency* of endodontically treated roots with zero to five defects for each group; and
(c) the frequency* of the different defects found in each group. (*Frequency is the percent ratio between the number of times a given defect is observed with respect to the total number of investigated samples [48]).

**Figure 2:**
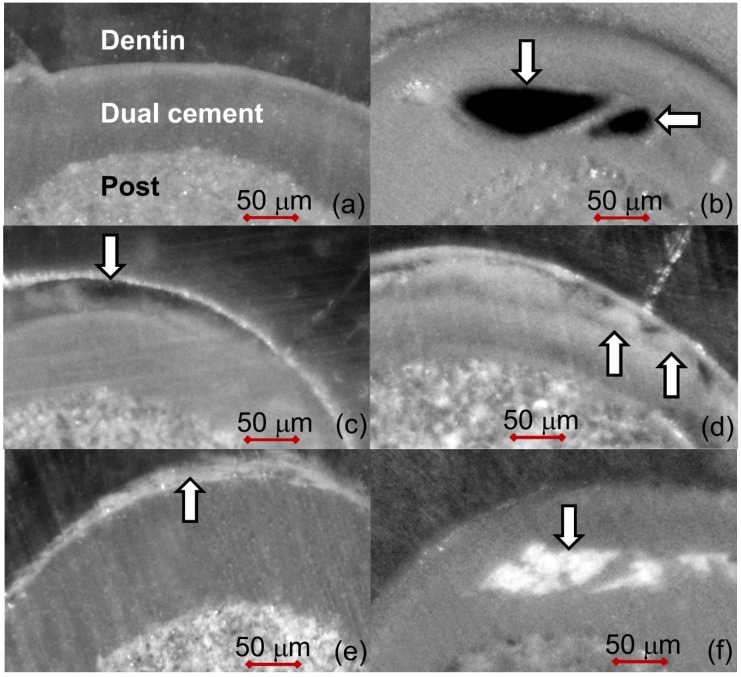
Seventy-micrometer thick unstained sections (enlargement of 40×): White arrows indicate the five defects detected: (**a**) defectless slice; (**b**) bubbles within the dual cement layer; (**c**) detachments at the dual cement-dentin interface; (**d**) polymerization defects within the dual cement layer (pol.def.); (**e**) endodontic cement residues at the dual cement-dentin interface (int.res.); and (**f**) embedded residues of endodontic cement into the dual cement layer (emb.res).

The extension of the single defects was evaluated on contact microradiographs to better identify their outline. The sections were then microradiographed at 8 kV and 14 mA by using a x-ray generator (XRG-3000, Ital Structures Research, Riva Del Garda, Italy). Contact microradiographs (High Resolution Film, Kodak) were developed (HC-110, Kodak), fixed (Hypam Rapid Fixer, Ilford), washed in bidistilled water and air dried at room temperature. Morphometric analyses were carried out on microradiographs by means of a light microscope and an image processing software (Nikon Nis-Elements BR). The defects’ dimensions were expressed in terms of percent area with respect to the whole dual cement area (Figure 2).

### 2.3. Statistical Analysis

Categorical data were compared using Chi-squared (χ^2^) with Yates’ correction or Fisher’s exact tests (Epi Info 3.5.3). Continuous data were expressed as mean ± standard error (Std.Err.) and compared using Anova and Bonferroni’s Multiple Comparison post Test (PRISM^®^ version 5.0, GraphPad Software, San Diego, CA, USA). In all comparisons, a p value <0.05 with 95% confidence intervals was considered statistically significant.

## 3. Results

In all the sections, the fiber-post appeared wholly surrounded by an endodontic cement layer. Sometimes apical sealant (AP or PCS) residues, both at the dentin-dual cement interface and within the dual cement, were found. Polymerization defects, bonding defects and gas bubbles embedded inside the dual cement were also detected.

### 3.1. Frequency of Defects

**Figure 3:**
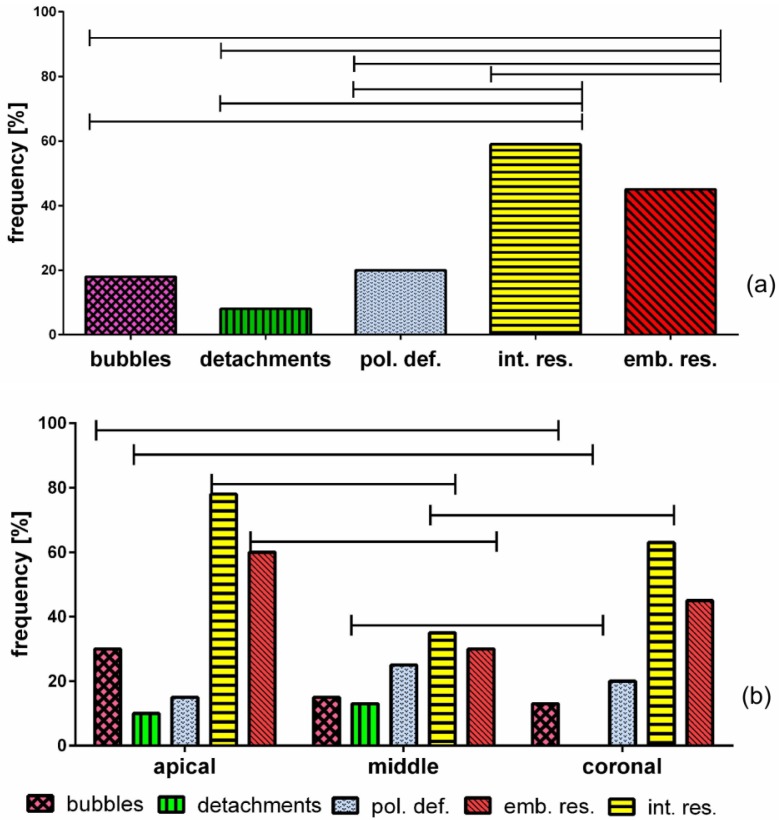
Frequency of defects taking all the sections together (regardless of either single specific level and irrigant/sealer combination) (**a**) and frequency of different defects in the three levels of the root (neglecting only the irrigant/sealer combination) (**b**). (pol.def = polymerization defects; int.res = endodontic cement residues at the dual cement-dentin interface; emb.res = embedded residues of endodontic cement into the dual cement layer).

Taking all together, the sections (*i.e.*, neglecting either the specific level and the irrigant/sealer combinations) sealant residues coating the dual cement-dentin interface were the most present defect (Figure 3a). Their frequency appeared significantly higher in respect to all the other defects (int.res. *vs.* bubbles and detachments p = 0.01, *vs.* pol.def. p = 0.02, *vs.* emb.res. p = 0.039 χ^2^), followed by sealant residues embedded within the dual cement (emb.res. *vs.* bubbles and pol.def. p = 0.02 χ^2^, *vs.* detachments p = 0.01 χ^2^), whereas the remaining ones were poorly represented.

Regardless of irrigant/sealer combination, statistically significant differences of some defects’ frequencies were found among the three levels investigated (Figure 3b).

In detail, gas bubbles were more frequently distributed in the apical than in the coronal sections (p < 0.05 χ^2^). No detachment was detected at the coronal level (coronal *vs.* middle p < 0.03, *vs.* apical p < 0.05 χ^2^). The frequency of residues at dentin-dual cement interface was lower at the middle level when compared with the other ones (middle *vs.* coronal p < 0.02, *vs.* apical p < 0.01 χ^2^). The endodontic sealant residues within the dual cement layer were significantly less represented at the middle level than at the apical one (p < 0.02 χ^2^). Regarding the polymerization defects, no statistically significant differences were found among the three levels.

**Figure 4:**
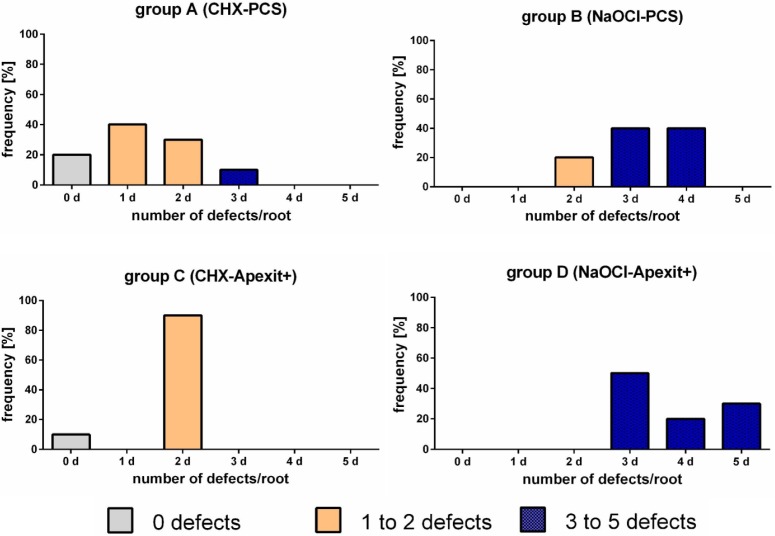
Frequency of endodontically treated roots with zero to five coexisting defects in each group.

The analysis of the coexistence of the five defects in every single group (Figure 4) showed many differences in relation to irrigant/sealer combination. In group A, 60% of the roots had one or no defect, whereas in the remaining 40%, two or almost three types of anomalies were found. In group C, except for 10% abnormality-free roots, two defects were always present.

On the contrary, in groups B and D, all the roots exhibited the simultaneous presence of 2 to 5 defects and none were defectless.

**Figure 5:**
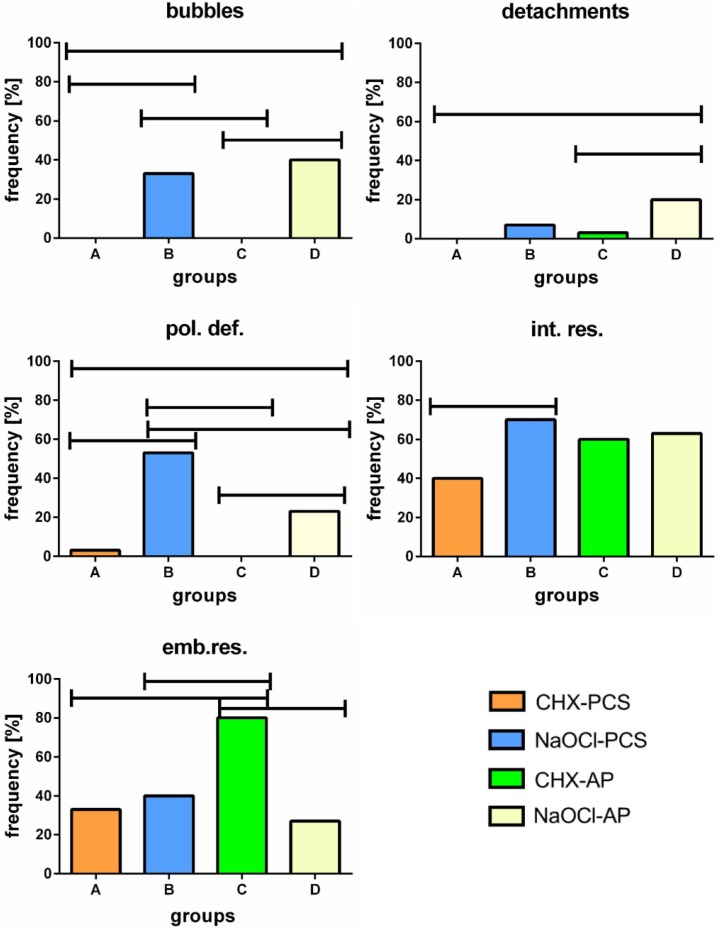
Frequency of the different defects found in each group. (pol.def = polymerization defects; int.res = endodontic cement residues at the dual cement-dentin interface; emb.res = embedded residues of endodontic cement into the dual cement layer).

Categorical data showed significant differences in the frequency of the various defects analyzed and the samples of groups B and D appeared to be the most damaged (Figure 5). In particular, gas bubbles were detected only in groups B and D (B *vs.* A and C, D *vs.* A and C p < 0.01 χ^2^). The detachment between dual cement and dentin was mainly found in group D (D *vs.* A p = 0.01, *vs.* C p < 0.05 Fisher), whereas the polymerization defects were mostly present in group B (B *vs.* A, *vs.* C p < 0.01 Fisher, *vs.* D p = 0.03 χ^2^). The latter defect was also detected in group D (D *vs.* A p < 0.03, *vs.* C p < 0.01 Fisher, *vs.* B p = 0.03 χ^2^).

Deposits of endodontic sealer along the dual cement-dentin interface were found in all the groups with a higher incidence in group B (B *vs.* A p = 0.038 χ^2^).

Finally, embedded residues within the dual cement seemed to be mainly present in the group C (C *vs.* A, *vs.* B and *vs.* D p < 0.01 χ^2^), whereas there were no statistically significant differences among the remaining groups.

### 3.2. Morphometric Analysis

**Figure 6:**
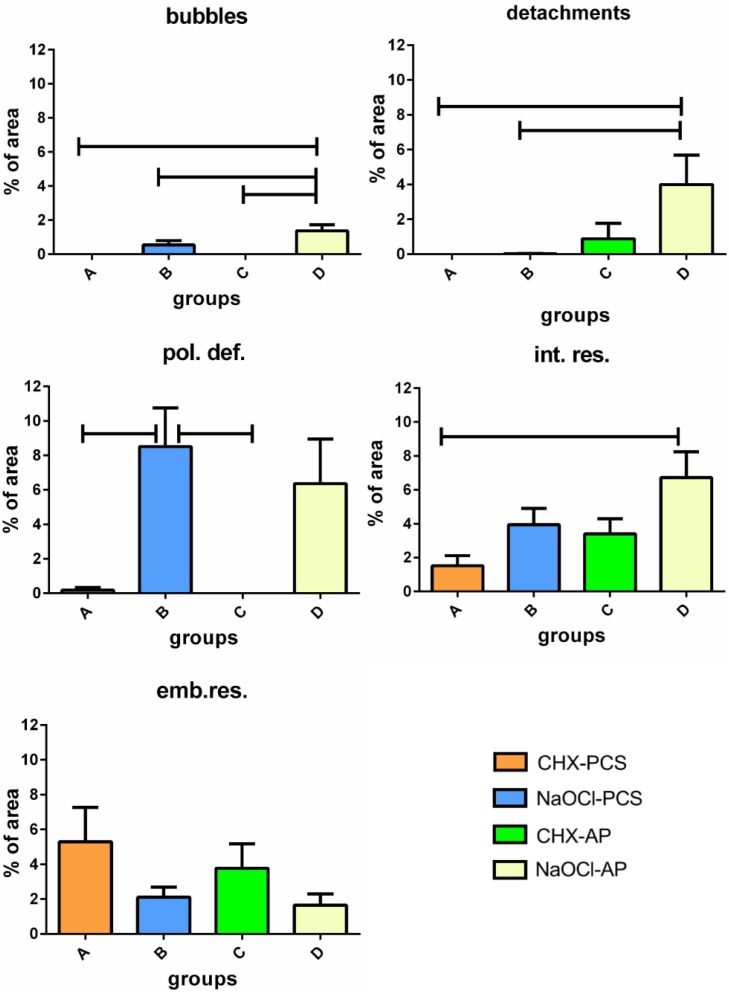
Mean (±Std. Err) percent area of each type of defect computed for the four groups. (pol.def = polymerization defects; int.res=endodontic cement residues at the dual cement-dentin interface; emb.res=embedded residues of endodontic cement into the dual cement layer).

Morphometric analyses revealed that the mean percent area (MPA) occupied by each defect does not exceed 9% and changes in relation to the type of defect and group (Figure 6). In particular, the highest values of MPA were found in group D for bubbles (p < 0.0001 Anova. D *vs.* A and C *vs.* D p < 0.01; *vs.* B p < 0.05 Bonferroni), detachments (p = 0.01 Anova. D *vs.* A and B p < 0.05 Bonferroni) and residues at the dentin-dual cement interface (p = 0.007 Anova. D *vs.* A p < 0.02 Bonferroni).

Wide areas of the dual cement layer were affected by polymerization defects mainly in groups B and D, whereas they seemed to be negligible in groups A and C (p = 0.0005 Anova. B *vs.* A and *vs.* C p < 0.02 Bonferroni).

Finally, no statistically significant differences about the embedded residues into the dual cement were found among the groups.

## 4. Discussion and Conclusions

The findings obtained in this study are consistent with those reported in the literature. Apical sealants (Apexit Plus or Pulp Canal Sealer) residues were detected at the dentin-dual cement interface as well as within the dual cement. These defects are the most frequent in all the sections analyzed, probably due to a failure in canal cleansing. In fact, the action of the drill used to remove the root filling material in order to create a post-space generates a new additional and thicker smear layer, which is rich in debris and sealer/gutta-percha remnants, plasticized and made gluey by the frictional heat. This smear layer may reduce the penetration and the chemical action of the bonding agents [49,50].

Detachments of dual cement from dentin were totally absent in the coronal third but they were observed in the apical and middle ones. This could be explained analyzing both: (i) the geometric factor and the polymerization shrinkage; and (ii) the type of the hybrid layer in each third of the root.

Regarding point (i), polymerization shrinkage is an unavoidable adverse effect occurring in resin-based materials. The polymerization process decreases the intermolecular spaces between the monomers and produces sufficient shrinkage stresses to debond the composite from dentin, thereby decreasing retention through gap formation [51]. Shrinkage stress can be related to the cavity configuration factor (C-factor) that is defined as the ratio of bonded to unbonded surfaces of the restoration: the lower the C-factor, the higher the capacity to dissipate polymerization shrinkage stresses and then the lower the probability of debonding [52,53,54]. A high C-factor characterizes the apical and middle levels, unlike a lower C-factor at the coronal level, where the sidewalls only are bonded, thus excluding the upper surface, made free after dental crown removal.

Glass ionomer (GIC) and resin modified glass ionomer cements (RMGIC) could be an alternative for fiber post cementation to avoid adverse effects of polymerization shrinkage of resin cements. A Pereira study revealed how GIC showed significantly higher values, in term of bond strength, compared to dual-polymerizing resin cements [55]. GIC and RMGIC show a hygroscopic expansion, which offset the initial shrinkage and increase the stability at the dentin-cement interface because of an increase frictional strength. Furthermore, they offer long lasting protection against dental caries by means of fluoride release [3,56,57,58].

With regards to aspect (ii), most of the studies state that bond strength to intra-canalar dentin decreases from the coronal to the apical third and that the thickness of the hybrid layer is considerably reduced in the apical third, causing a weaker bond strength because of the reduced impregnation of the adhesive system [59]. The better performance in the coronal region is also ascribable to the simple approachability of this part of the canal space [59], making etching and adhesive procedures easier [60].

Endodontic sealers play an important role in the post retention. Only few studies about the influence of calcium hydroxide sealers on resin polymerization and post retention were conducted. A recent study by Demiryürek showed that specimens with calcium hydroxide-based sealer revealed the highest bond strength if compared with resin-based and zinc oxide–eugenol-based sealer [61]. In the present study, no resin-based sealers were used as endodontic sealer because they penetrate deeply into dentinal tubules thus decreasing the bond strength of resin cement [61,62]. Eugenol-based sealers remain the primary choice for endodontics because of their long history of clinical success [63]. However, eugenol can infiltrate dentin [36,39,40], thus reducing the bonding efficiency and causing polymerization defects [41]. This aspect is consistent with the results reported in this study. In fact, samples treated with NaOCl show a higher frequency of polymerization defects when associated to PCS than to AP. Furthermore, since these defects are overall more frequent in groups B and D (Figure 5), it is possible to hypothesize that they are more sensitive to the irrigant than to the cement.

Furthermore, samples treated with NaOCl show higher frequencies of bubbles and detachments (Figure 5 and Figure 6), thus confirming a dominant role of irrigants over cements.

Sodium hypochlorite has been considered an excellent non-specific proteolytic agent that dissolves organic compounds [64,65], enhancing the penetration of composite monomers into the demineralized dentin structure [66]. Despite its positive effects, NaOCl can cause problems if used in association with resin-based materials due to its strong oxidizing properties, leaving a dentin surface coated by an oxygen-rich layer. Oxygen interferes with resin infiltration into the tubules and intertubular dentin [29,30,31,32,66]. This might partially explain why samples of groups B and D exhibit the largest frequency and extension of bubbles and detachments when compared with groups A and C (Figure 5 and Figure 6). The use of 37% phosphoric acid as etching agent has been reported to remove remnants of the eugenol incompletely and of the contaminated smear layer [67]: This leads to the demineralization of dentin to a depth of 9–10 μm [68]. This depth of demineralization and the water rinsing after etching reduces the amount of free eugenol on the dentin surface [38]. The higher amount of bubbles in the apical than in the coronal sections might be due to the difficulty of the etching gel to reach the post-space end during the preparation procedure of the specimens. The lack of the etching gel could bring about the persistence of an oxygen-rich layer in the post space apical dentin. However, the use of 37% of phosphoric acid for 60 s increased the bond strength in the apical third of the root dentin [69].

Finally, CHX seems to induce a lower number of abnormalities when compared with NaOCl. In fact, only within groups A and C, defectless sections were found. Conversely, the number of coexisting defects in a given sample appears to be higher when using NaOCl (Figure 4).

In conclusion, various irrigation/sealer combinations appear to produce differences in the frequency, type and extension of defects. CHX seems to confirm its higher capability of increasing the luting agents adhesion to dentin in respect to NaOCl. In addition, when CHX is associated with PCS, it seems to produce a significant reduction in defects. However, the data obtained in this study do not allow determining how much these differences may affect the fiberglass endopost stability. The correlation between frequencies and extension of the five detected defects and the endopost’s bond strength will be the purpose of a forthcoming research.
